# Different patterns of activated partial thromboplastin time (APTT) clot waveforms predicting the presence of lupus anticoagulant

**DOI:** 10.1016/j.clinsp.2026.101136

**Published:** 2026-09-10

**Authors:** Kazutoshi Ebisawa, Masaki Tanibuchi, Kunitoshi Fukasawa, Takahiro Takeuchi

**Affiliations:** a Department of Hematology, Shizuoka Saiseikai General Hospital, 1-1 Oshika, Suruga-ku, Shizuoka, Japan; b Department of Laboratory medicine, Shizuoka Saiseikai General Hospital, 1-1 Oshika, Suruga-ku, Shizuoka, Japan

**Keywords:** Lupus anticoagulant, Antiphospholipid syndrome, APTT, Clot wave analysis, Clot waveform

## Abstract

**Introduction:**

Atypical peak pattern in second derivative curve of clot wave of Activated Partial Thromboplastin Time (APTT) was known to be associated with presence of Lupus Anticoagulant (LA), but clinical significance of other abnormal patterns was uncertain.

**Method:**

We retrospectively reviewed second derivative curves of clot waves of 271 samples with prolonged APTT (> 50 *sec*). These were approximated by fifth-order functions using polynomial regression analysis, and Principal Component Analysis (PCA) was performed to classify these functions.

**Results:**

PCA analysis could separate a cluster of abnormal waveforms from that of normal pattern. Abnormal waveforms could be classified into atypical peak pattern, S-shaped pattern and Left Bundle Branch Block (LBBB)-like pattern, which formed discrete clusters on the PCA plot. While only a small proportion of cases with normal pattern were positive for LA (8/202 cases), all of these abnormal patterns were associated with LA (5/23, 9/27, 4/19 cases, respectively).

**Discussion:**

Our analysis demonstrated that as well as atypical peak pattern, S-shaped and LBBB-like patterns could predict the presence of LA in cases with prolonged APTT.

## Highlights

• We performed PCA after approximation of 2nd derivative curves of APTT.
• Three different abnormal waveforms were detected by this PCA.
• All the abnormal waveform patterns were associated with the presence of LA.
• This predictive performance was highest when APTT was <60 s.

## Introduction

Prolonged Activated Partial Thromboplastin Time (APTT) was one of the common abnormalities of coagulation system, whose etiology included hemophilia, von-Willebrand disease, vitamin K deficiency, effect of anticoagulant drugs such as heparin, and presence of Lupus Anticoagulant (LA).^1^ While most of the differential diagnoses were categorized into bleeding disorders, presence of LA showed thrombotic rather than bleeding tendency. Accurate diagnosis was crucially important, but confirmation tests of LA usually required several days. In addition, there were several types of LA tests depending on the reagents such as diluted Russell’s viper venom, and detection of LA might or might not be possible depending on the types of reagents used.^2^

Clot waveform of APTT was automatically attained when measuring APTT, which reflected dynamics of fibrin clot formation.^3^ There had been several researches on its clinical usefulness, such as assessment of the severity of coagulopathies induced by infections[4–6] or acute promyelocytic leukemia,^7^ prediction of bleeding risks,^8^^,^^9^ monitoring of treatment for patients with hemophilia A,^10^, ^11^, ^12^ detection of LA,^13^ or assessment of hemostatic activity under various conditions including malignant neoplasms,^14^ cerebral and myocardial infarctions,^15^^,^^16^ abdominal surgery^17^ or idiopathic thrombocytopenic purpura.^18^ Previous studies indicated that abnormal pattern with atypical peak observed in the second derivative curves was associated with the presence of LA,^13^^,^^19^ but whether other abnormal visual patterns were associated with the presence of LA were not studied well. Here, we retrospectively analyzed more than 250 samples with prolonged APTT, and revealed that abnormal patterns other than atypical peak were associated with the presence of LA.

### Patients

The results of APTT tests measured in our hospital from 7/1/2024 to 12/31/2024 were retrospectively reviewed, and those with prolonged APTT to 50-seconds or longer were included in our analysis. As same patient might have different waveform patterns, duplication was permitted. Pediatric patients were not included. APTT was measured with automated blood coagulation analyzer CS-2500 or CS-5100 (Sysmex Corporation, Kobe, Japan) using Revohem APTT SLA reagent (Sysmex Corporation). Clinical information of patients was collected from their medical records. This study was performed in accordance with the principles of the Declaration of Helsinki and was approved by the Ethics Review Board of our institute (Approval n° 20,250,605). The requirement for written informed consent was waived because of the retrospective nature of the study, and an opt-out approach was used in accordance with institutional policy. In addition, this study was conducted and reported in accordance with the Strengthening the Reporting of Observational Studies in Epidemiology (STROBE) Statement.

### Statistical analysis

**Fig. 1:**
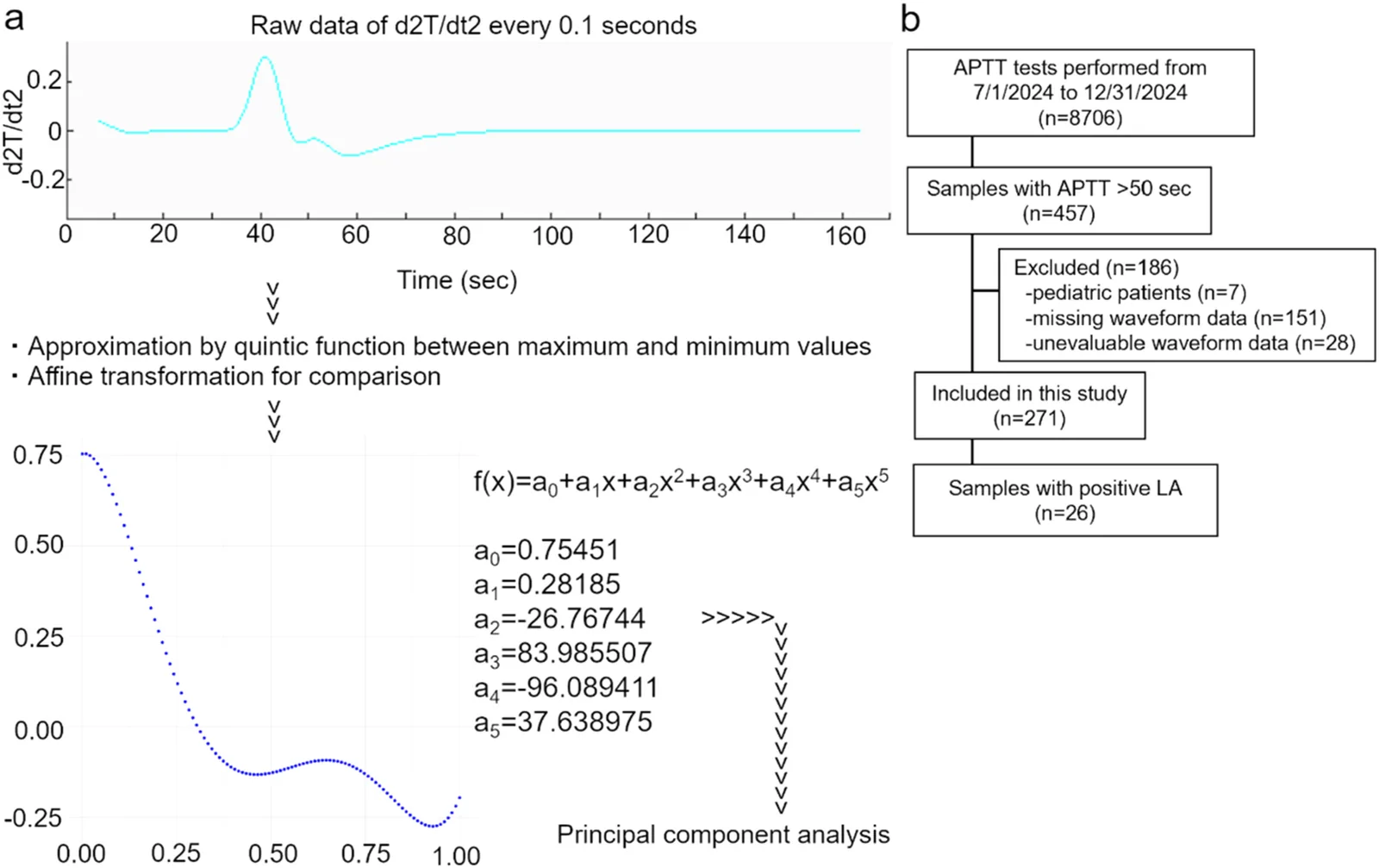
**Scheme of this study.** (a) Raw graph data of a representative case with atypical peak pattern and the graph after approximation by quintic function and affine transformation were shown. Coefficients of this function was shown, but it should be noted that these were rounded for ease of understanding. In addition, a flow diagram of this study (b) was shown.

Numerical variables between groups were compared with Kruskal-Walis tests, and difference of categorical variables were compared with Chi-Square tests. Concomitant with routine APTT measurement, values of absorbance were recorded every 0.1 seconds automatically, and values of first and second derivatives corresponding to each time point were also calculated. Curves of second derivative were also automatically depicted. After identifying maximum and minimum values of second derivative curves and the time points that gave them, polynomial regression analyses were performed at these intervals using the least square method. As the x-axis values (time) which gave the maximum and minimum values varied greatly, affine transformation was performed such that the point giving the maximum value was (1.0) and the point giving the minimum value was (0.1), intending to compare the shape of the curves itself. Principal Component Analysis (PCA) was performed using the sets of coefficients of the quintic functions obtained in this way. Scheme of this process was summarized in Fig. 1. Next, κ-means clustering was subsequently applied to the PCA scores. The optimal number of clusters was evaluated using the Gap statistic, with four clusters selected because the Gap statistic showed its first decline at κ=4. Accordingly, the dataset was partitioned into four clusters. Waveforms of all cases were manually classified. Waveforms which had clear local maximum value were classified into atypical peak pattern. S-shaped pattern was defined as waveforms whose amount of change temporarily decreased at around the midpoint between the maximum and minimum values, but did not turn into an increase and returned to its original amount of change. If the amount of change decreased at around the midpoint, and then continued to decrease at this rate until it reached to the minimum value, such waves were classified into LBBB-like pattern. Other waves were classified into normal pattern. KE and other three doctors independently classified the waveforms, and inter-rater reliability was assessed by calculating Fleiss’ kappa value. Multivariate logistic regression analysis was performed to evaluate association between abnormal waveforms and each variable. Variables showing a p-value <0.10 in univariate analysis were considered as candidates for multivariate analysis, and clinical importance of each variable was also considered in generating the final model. Diagnosis of infection was based on imaging studies (e.g., pneumonia or intra-abdominal infection), blood or urine tests (e.g., pancreatitis or urinary tract infection), or direct microbiological tests of the pathogen (e.g., COVID-19) regardless of etiology of prolonged APTT. The cutoff value of APTT (60-seconds) was based on previous reports.^20^^,^^21^ All the statistical analyses were performed using R 3.6.0. In all the analyses, the level of significance was defined as two-tailed p-value of less than 0.05. No part of this article was created with assistance from Artificial Intelligence (AI) tools.

## Results

### Identification of abnormal waveform patterns by polynomial regression and PCA analysis

**Fig. 2:**
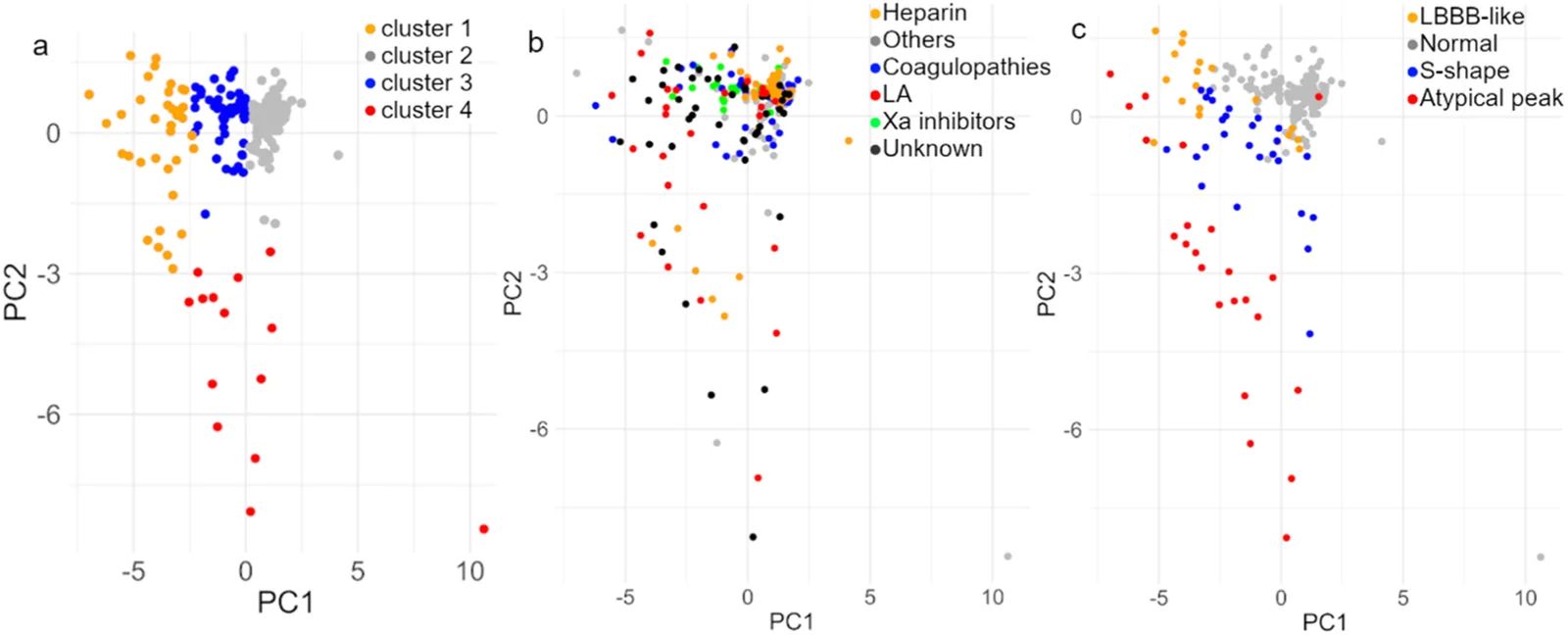
**Results of Principal Component Analysis (PCA).** Results of PCA in which each plot was classified according to (a) Clusters identified by κ-means clustering method, (b) Etiologies of prolonged APTT and (c) Clot waveforms. Causes of coagulopathies in Fig. 2b were massive bleeding (*n* = 11), malignant diseases (*n* = 3), infectious diseases (*n* = 32), infectious + oncologic diseases (*n* = 3), and others (*n* = 5).

Among the 457 samples with APTT >50 seconds, seven samples from pediatric patients were excluded. Clot waveform data was available in 299 cases. Twenty-eight cases were excluded because appropriate coagulation waveforms could not be obtained (Fig. 1b). Polynomial regression analysis was performed for each second-order derivative (d2T/dt2) curve between maximum and minimum values. Since there was quite a large variation in the x-axis values corresponding to the maximums and minimums, affine transformation was performed to minimize the effect of this difference on the results of the following PCA analysis, which intended to compare the shape of the clot waveform itself. PCA analysis identified four different clusters (Fig. 2a). LA-positive samples were predominantly distributed in clusters 1 and 4, whereas they were rarely observed in cluster 2 (Fig. 2b, Table 1).

**Table 1:** **Results of clustering of patients in this study.** In the principal component analysis, four different clusters were identified by κ-means clustering method. Brief clinical characteristics, presence of lupus anticoagulant and clot waveform patterns were shown for each cluster.

		Cluster 1	Cluster 2	Cluster 3	Cluster 4	p-value
**Age**		77 (66‒84)	81 (66‒89)	78 (67‒82)	69 (66‒83)	0.3
**APTT**		68.4 (57.8‒86.5)	59.0 (53.9‒73.1)	68.2 (55.5‒84.2)	113.1 (106.4‒123.0)	<0.01
**Machine**	CS2500 / CS5100	16/21	95/69	31/25	7/7	0.423
**Gender**	Female / Male	18/19	79/85	37/19	4/10	0.0364
**LA**	Positive/Negative	13/24	6/158	3/53	4/10	<0.01
**Clot waveform**						<0.01
	Normal	2	156	43	1	
	S-shape	11	3	11	2	
	LBBB-like	13	4	2	0	
	Atypical peak	11	1	0	11	

LA; Lupus Anticoagulant.

**Fig. 3:**
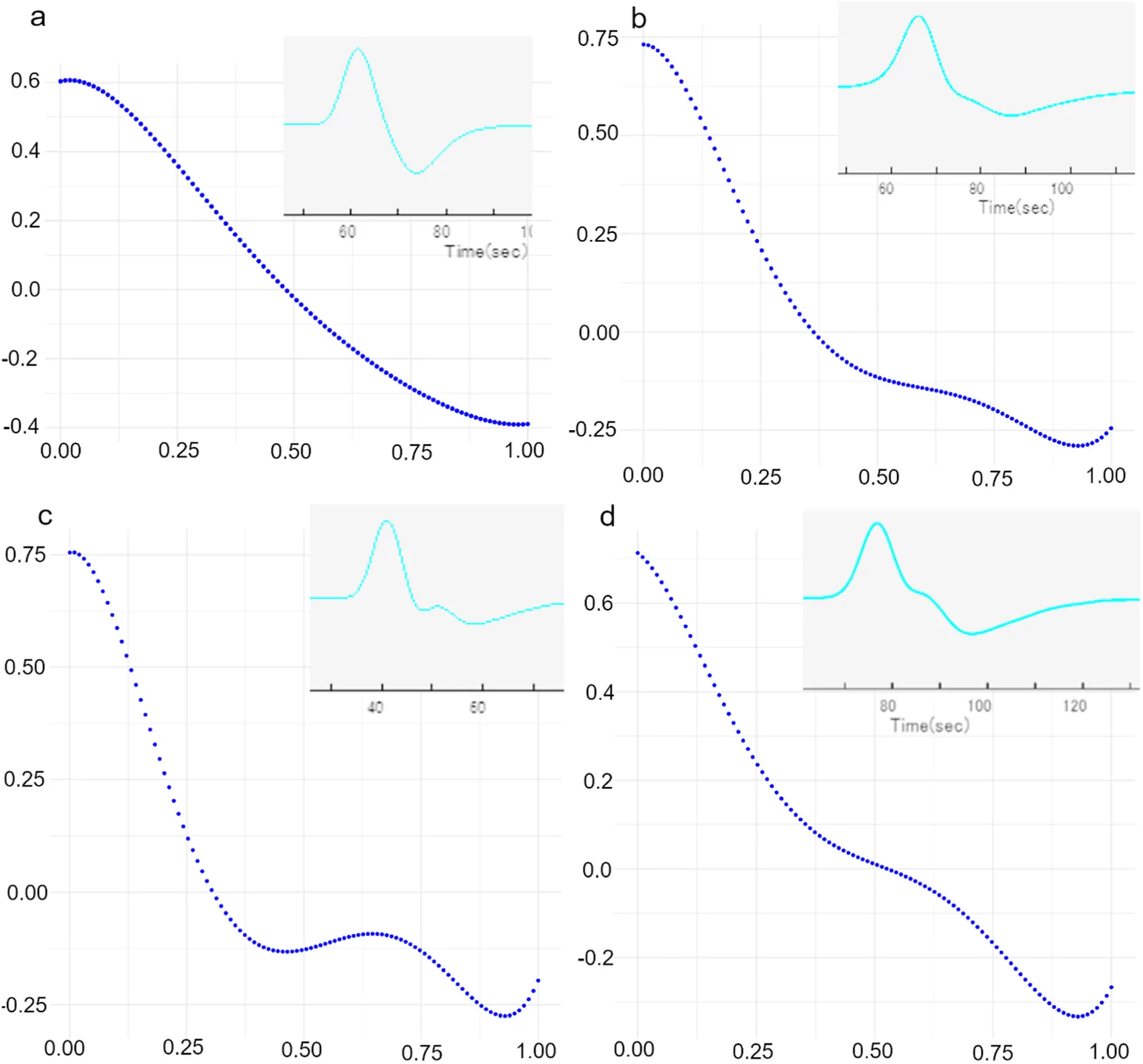
**Scheme of abnormal waveform patterns.** Second-order derivative curves and graphs after approximation by quintic function and affine transformation were show for representative samples with (a) Normal pattern, (b) LBBB-like pattern, (c) Atypical peak pattern and (d) S-shaped pattern. For ease of viewing, second-order derivative curves were extracted from the raw data.

Next, waveforms of all the 271 samples were manually analyzed. Among 51 samples in cluster 1 and 4, in addition to cases with typical “atypical peak” pattern (22/51), there were as many cases with no abnormal peaks, but with a sharp decrease in the rate of decrease in the middle of the curves. As shown in Fig. 3, these waveforms could be divided into two patterns: one with an S-shaped waveform (S-shaped pattern, 13/51) and the other with a waveform similar to the V6-induced Electrocardiogram (ECG) waveform observed in complete Left Bundle Branch Block (LBBB-like pattern, 13/51). Based on this classification, 202, 23, 19 and 27 samples were classified into normal, atypical peak, LBBB-like and S-shaped pattern (Table 2). Inter-rater reliability test was performed to validate this classification, indicating substantial agreement among raters with Fleiss’s kappa-value of 0.775.^22^ Samples with atypical peak pattern were predominantly distributed in the PC2-negative region of cluster 1 and cluster 4, and samples with LBBB-like pattern were predominantly located in PC2-postivie region of cluster 1. Samples with S-shaped pattern were seen in clusters 1 and 3, and most samples with normal patterns were included in cluster 2. These positional relationships strongly suggested that waveforms seen in atypical peak and LBBB-like patterns were clearly different from the normal waveform, and waveforms of S-shaped patterns were “intermediate” forms between these patterns.

**Table 2:** **Clinical characteristics of patients in this study.** Clinical information on all samples was shown, permitting multiple samples from single patient.

		Normal	Atypical peak	LBBB-like	S-shaped	p-value
**Age**		80 (67‒88)	71 (66‒81)	76 (54‒90)	80 (59‒84)	0.47
**APTT**		60.0 (53.8‒74.4)	102.6 (60.6‒111)	68.4 (56.4‒80.1)	84.3 (60.1‒106.9)	<0.01
**Machine**	CS2500 / CS5100	118 / 84	9 / 14	9 / 10	13 / 14	0.2355
**Gender**	Female / Male	106 / 96	6 / 17	12 / 7	14 / 13	0.29
**Etiologies**						<0.01
	LA	8	5	4	9	
	CPA	10	0	0	2	
	Hemophilia A	0	1	0	0	
	Liver disease	11	1	0	1	
	Drug-induced					
	- Heparin	61	6	1	0	
	- Thrombin inhibitor	5	0	0	0	
	- Warfarin	11	0	4	0	
	- Xa inhibitors	28	1	2	1	
	Coagulopathy by infection^a^	28	1	0	3	
	Other coagulopathies					
	- Massive bleeding	10	0	0	1	
	- Oncologic DIC	1	2	0	0	
	- Infectious^b^ + oncologic	3	0	0	0	
	- Other etiologies	5	0	0	0	
	Unknown	21	6	8	10	

a Among the 32 cases, 21 had pneumonia, 3 had urinary tract infection, 3 had intra-abdominal infection, and 2 had COVID-19. There was one patient with pancreatitis, blood stream infection, and cholecystitis, respectively.

b All the three cases had pneumonia. LA, Lupus Anticoagulant; CPA, Cardiopulmonary Arrest; DIC, Disseminated Intravascular Coagulopathy.

Among the 28 samples with unevaluable waveforms (Fig. 1b), APTT levels were prolonged to more than 120 seconds in 27 samples, and 150 seconds in 21 samples. Reasons for difficulty in evaluation were second-derivative curves that broke off midway (*n* = 24) and chaotically shaped curves (*n* = 4). Eleven samples were attained from patients included in the analysis above. The etiologies were LA (*n* = 5), heparin (*n* = 5) and coagulopathy from uncontrolled leukemia and infection (*n* = 1). The etiologies of the resting 17 samples were LA (*n* = 1), heparin (*n* = 6), warfarin (*n* = 1), coagulopathy by infection (*n* = 2), CPA (*n* = 3), congenital factor XII deficiency (*n* = 1) and unknown (*n* = 3) (Supplementary Table 1).

### Association between etiology and pattern of waveform

**Fig. 4:**
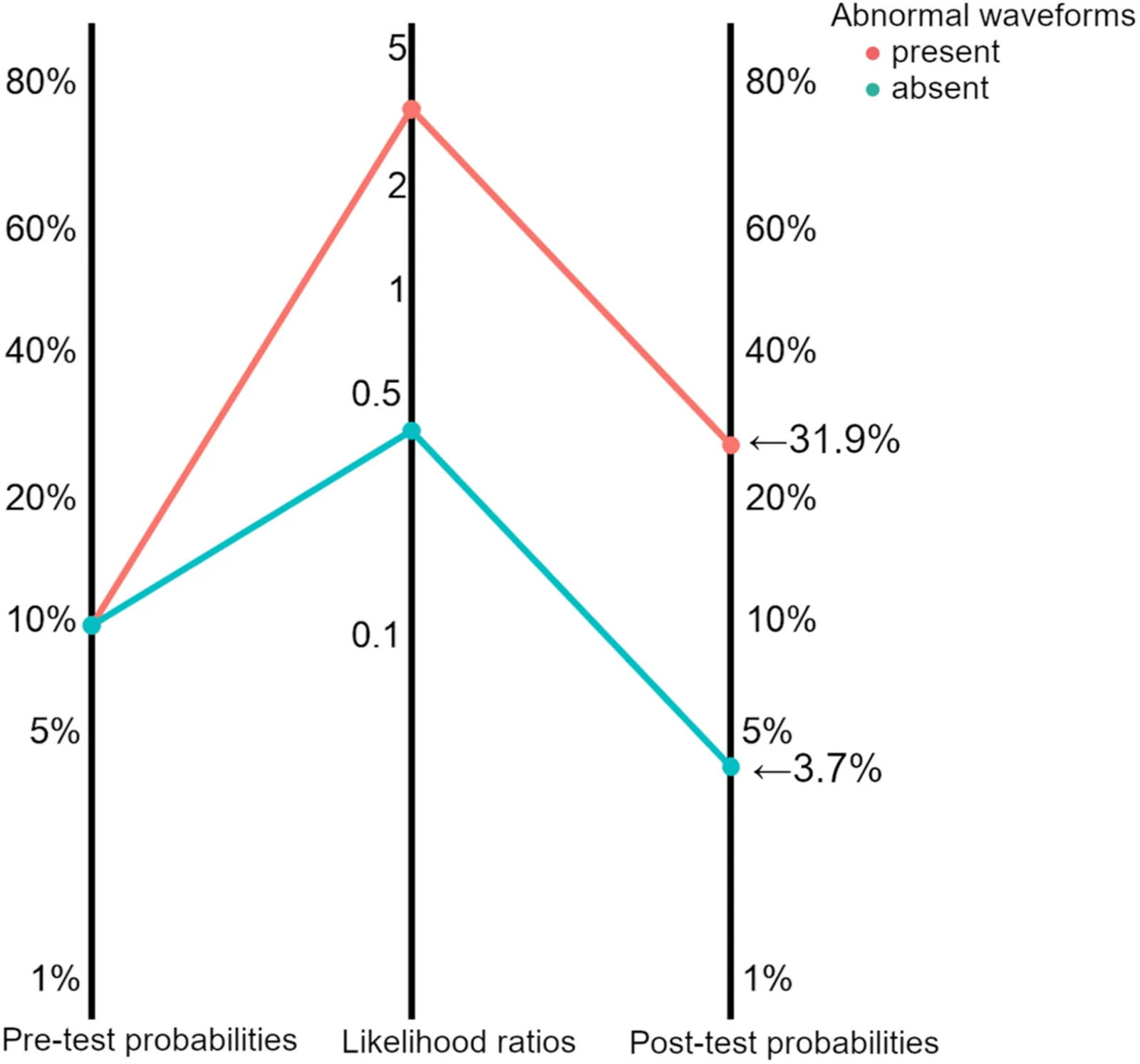
**Predictive performance of abnormal clot waveforms.** Fagan nomogram illustrating the pre- and post-test probabilities of presence of lupus anticoagulant according to the clot waveform patterns. Pre- and post-test probabilities were shown in the left and right axes, and likelihood ratios of each clot waveform patterns were shown in the middle axis. The pre-test probability was based on the sample prevalence in our patients.

Cases with LA were more frequently classified into one of the abnormal waveform patterns (18/69, 26.1%) than the normal pattern (8/202, 4.0%), which corresponded to Predictive Value (PPV) of o 26.1% (IQR: 16.3‒38.1%) and Negative Predictive Value (NPV) of 96.0% (IQR: 92.3‒98.3%), respectively. Positive and negative Likelihood Ratios (LRs), which were independent of prevalence rate, were 3.326 (IQR: 2.334‒4.378) and 0.389 (IQR: 0.218‒0.694), respectively (Fig. 4). As for each waveform pattern, positive and negative LRs were 2.618 (IQR: 1.059‒6.468) and 0.872 (IQR: 0.72‒1.055) for atypical peak pattern, 2.513 (IQR: 0.901‒7.012) and 0.901 (IQR: 0.763‒1.065) for LBBB-like pattern, and 4.712 (IQR: 2.362‒9.398) and 0.706 (IQR: 0.532‒0.935) for S-shaped pattern, respectively.

Further, out of concern for potential effects of anticoagulants^23^ and infectious diseases^5^ on clot waveforms, multivariate logistic regression analysis was performed (Table 3). There were 87 samples with infectious diseases, including 51 samples with pneumonia, 13 with urinary tract infection, 10 with intra-abdominal infection, 6 with COVID-19, and 3 with pancreatitis. 11 samples had abnormal waveforms, but no patients had positive LA. Heparin, warfarin, and DOACs were used in 70, 20, and 44-samples, respectively, whereas the crude ORs for warfarin and DOACs did not reach statistical significance in the univariate analysis. The Events Per Variables (EPV) of this model was 11.5, and adjusted OR of LA in the final model was 4.69, but that of prolonged APTT (>60-seconds) was also significant. In this cohort, APTT was more prolonged in LA-positive patients compared to LA-negative patients (81.2 vs. 61.3 seconds, p < 0.01). Since the more prolonged the APTT, the easier it could be for raters to detect waveform abnormalities, concerns arose that LA itself might not be the direct cause of the waveform abnormalities, but rather that the excessive prolongation of APTT simply makes it easier to detect them. In fact, additional multivariate model which included an interaction term (LA*APTT > 60 seconds) demonstrated a significant interaction between them (OR = 0.071, p = 0.034). Stratified analyses demonstrated that the association between LA positivity and abnormal patterns was stronger among patients with APTT < 60 seconds (adjusted OR = 17.1, p = 0.013) than among those with APTT ≥ 60 s (adjusted OR = 3.3, p = 0.042).

**Table 3:** **Logistic regression analysis for presence of abnormal waveforms.** Results of univariate and multivariate logistic analysis for the presence of abnormal waveforms were shown. Analysis stratified by APTT of 60-seconds was also shown.

	Univariate analysis		Multivariate model	
**Variables**	**Crude odds ratio (95% CI)**	**p‒value**	**Adjusted odds ratio (95% CI)**	**p‒value**
Lupus anticoagulant: present vs. absent	8.47 (3.28‒23.85)	**<0.01**	4.69 (1.76‒12.50)	**<0.01**
Age: >66 vs. ≤66	0.50 (0.27‒0.95)	**<0.028**	0.58 (0.13‒0.66)	0.133
APTT: >60 vs. ≤60 s	2.55 (1.42‒4.59)	**<0.01**	2.41 (1.27‒4.58)	**<0.01**
DOACs: Yes vs. No	0.61 (0.23‒1.42)	0.26		
Gender: Male vs. Female	1.28 (0.71‒2.30)	0.41	1.70 (0.90‒3.19)	0.10
Heparin: Yes vs. No	0.30 (0.12‒0.67)	**<0.01**	0.27 (0.11‒0.65)	**<0.01**
Warfarin: Yes vs. No	1.28 (0.39‒3.73)	0.60		
Infection: Yes vs. No	0.32 (0.14‒0.65)	**<0.01**	0.46 (0.22‒0.99)	**0.046**
	**APTT <60 s**		**APTT >60 s**	
**Variables**	**Adjusted odds ratio (95% CI)**	**p-value**	**Adjusted odds ratio (95% CI)**	**p-value**
Lupus anticoagulant: present vs. absent	17.1 (2.47‒345.41)	**0.013**	3.30 (1.07‒11.09)	**0.042**
Age: >66 vs. ≤66	0.51 (0.14‒1.88)	0.30	0.61 (0.25‒1.47)	0.27
Gender: male vs. female	0.55 (0.15‒1.88)	0.35	3.02 (1.40‒6.90)	**<0.01**
Heparin: Yes vs. No	Not estimable	‒	0.36 (0.13‒0.90)	0.036
Infection: Yes vs. No	0.12 (0.006‒0.68)	0.050	0.71 (0.28‒1.70)	0.45

DOAC, Direct Oral Anticoagulant.

Our analysis included 26 samples with positive LA from 9 patients. Notably, 9 and 7 tests each were attained from 2 patients, and as for the remaining 7 patients, only one to two samples each were included. Contrary to our expectation, even the samples taken from the same patient showed different abnormal waveform patterns (Supplementary Table 2). Among the 9 samples from one patient treated with heparin against catastrophic antiphospholipid syndrome, 5 samples showed normal waveform, other two were S-shape, and the other two were LBBB-like. The patient had a fulminant clinical course and died shortly after treatment initiation. Thus, only one sample after start of heparinization could be attained, which showed LBBB-like pattern. The 7 samples from another patient, who were positive for LA but complicated with coagulopathy from fatal gastrointestinal bleeding after orthopedic surgery, showed normal pattern in 3 samples, S-shape in other 3 samples and atypical peak in 1 sample. First two samples were attained when the patient took edoxaban, which showed relatively mild APTT prolongation with 51.1 and 65.2 seconds. While edoxaban was discontinued soon, APTT prolongation was rather worsened. Among the five samples attained after discontinuing edoxaban, 3 samples had S-shaped pattern, 1 sample had atypical peak pattern, and the other sample had normal pattern.

To minimize the clustering effects, analysis using first samples from each patient was performed (supplementary Table 3). While LA was positive just in 2 in 124 samples with normal waveform, 7 in 35 samples with abnormal waveforms was positive for LA. Presence of abnormal wave patterns gave similar positive and negative LRs, which were 4.167 (IQR: 2.57‒6.756) and 0.273 (IQR: 0.08‒0.93), respectively.

## Discussion

In our study, clot waveforms of 271 samples were comprehensively analyzed and compared using the objective method of approximation by polynomial equation. PCA analysis identified clusters which consisted of many cases with abnormal waveform patterns, and LA-positive cases were included in these clusters more frequently.

Further, we classified abnormal wave patterns into three patterns; atypical peak, S-shape and LBBB-like. Yatomi et al., evaluated association between clot waveform patterns and clinical outcome of patients with COVID-19, and classified abnormal patterns into biphasic type, early shoulder type and late shoulder type.^4^ Biphasic type corresponded to atypical peak in our analysis. Late and early shoulder type seemed to correspond to LBBB-like and S-shaped, although there might be some overlap. Their research demonstrated that clot waveform abnormalities were associated with severity of COVID-19, but whether the abnormal patterns were associated with LA was not evaluated well, possibly because of the limited number of samples with LA (*n* = 2, both were early shoulder type). Although previous studies revealed that atypical peak pattern could predict the presence of LA,^13^^,^^19^^,^^24^ predictive performance of abnormal clot waveforms other than atypical peak pattern had not been studied well. Recent study by Nithye et al., demonstrated that atypical shoulder pattern was associated with positive LA,^19^ but study by Solano et al., indicated that atypical shoulder pattern had only limited diagnostic performance.^24^ It was true that our “atypical peak pattern” and “S-shape pattern” seemed abnormal patterns previous studies had pointed out, but as far as we knew, analysis on waveforms categorized into “LBBB-like pattern”, which had neither abnormal peaks nor shoulders, had not performed yet.

We classified as many as 271 samples with prolonged APTT into three abnormal patterns, but our classification was based solely on visual findings. Whether such subjective classification was objectively valid had not been studied yet, so approximation of clot waveforms by fifth-order functions and PCA analysis were performed. These results strongly indicated that as well as atypical peak pattern, S-shaped and LBBB-like patterns were different from normal pattern, which would justify the visual classification we had performed.

In addition, our study demonstrated that as well as atypical peak pattern, S-shaped pattern and LBBB-like pattern could also predict the presence of LA. Although rigorous statistical analyses to identify which waveform abnormality was most predictive of the presence of LA could not be performed because of the limited sample size, it was also demonstrated that abnormal waveform patterns had high NPV, but concerning the impact of LA on treatment strategies, PPV of 20%‒30% would not be low. Because analysis of clot waveforms was rather a screening test for LA than a confirmation test, clinicians should perform confirmation tests of LA if patients with prolonged APTT had waveform abnormalities. Conversely, high NPV rate suggested that the confirmatory tests might be unnecessary if such patients did not have any waveform abnormalities and had obvious causes for APTT prolongation such as anticoagulant therapy.

In our PCA analysis, clusters of S-shaped pattern and LBBB-like pattern were located relatively close to the cluster of normal patterns compared to the cluster of atypical peak pattern. The cluster of atypical peak pattern was located furthest from the cluster of normal patterns, extending consecutively from the cluster of S-shaped pattern. This spatial relationship in the PCA plot was consistent with a finding of in the inter-rater analysis, in which identification of atypical peak pattern was straightforward, whereas some cases were difficult to distinguish between LBBB-like and S-shaped patterns. Furthermore, it was speculated that there were no fundamental differences between these three waveforms, at least between S-shaped pattern and atypical peak pattern, and all of them might reflect the same biological process. Under normal conditions, the second derivative curves decreased almost linearly between maximum and minimum values, but inhibitory substances such as LA slowed down the rate of decrease. If the degree of inhibition was strong enough, the curves would be inverted and showed atypical peak pattern, and if not so, the curves showed LBBB-like or S-shape pattern. Although basic physiological verification would be warranted, this hypothesis could explain why samples with atypical peak tended to be plotted at more distant areas from the cluster of normal patterns than the clusters of S-shaped and LBBB-like patterns.

Data of clot waveform was not available in approximately one-third of whole cases possibly because of failure in the process of data collection. Considering the nature of retrospective study, it could not be completely ruled out that sampling bias or confirmation bias might have been involved in the process of data collection. In addition, waveforms of twenty-eight samples were not appropriate for classification. APTT of these patients was extremely prolonged, and samples with positive LA were enriched in these population (21.4%). Therefore, it should be recognized as a limitation that, in groups with the most pronounced APTT abnormalities, waveform analysis itself might become impossible. In such cases, comprehensive investigation into the etiologies of prolonged APTT including LA should be performed.

Performed in a community hospital, there were cases in which treating physicians did not investigate the etiology of APTT prolongation or even consult to hematologists. In such cases, the etiologies were retrospectively speculated from their medical records, but we could not identify the etiologies in 43 samples. However, as most of these patients were not treated with anticoagulant agents, and did not have diseases like sepsis or liver diseases that could develop defect in coagulation systems, it would be reasonable to speculate that some of these patients might have been positive for LA if appropriately evaluated.

Although our study demonstrated that LA was a major cause of atypical waveforms with prolonged APTT, it must be acknowledged that conditions other than LA also caused abnormal waveforms. In fact, among 35 first samples with abnormal waveforms from each patient, anticoagulants were administered in 7-patients (DOAC; *n* = 4, warfarin; *n* = 2, heparin; *n* = 1), coagulopathies from infectious diseases and malignant disease were present in 3 and 1 patients, while LA was confirmed in 7 patients. These results were consistent with previous reports indicating that clot waveforms of APTT were influenced by infectious diseases,^4^, ^5^, ^6^ anticoagulant medicine,^23^ and oncologic diseases.^7^ In the multivariate analysis (Table 3), however, it was demonstrated that anticoagulants and infection were not independently significant predictors of abnormal waveforms in the presence of LA, which suggested that even when evident causes of APTT prolongation such as anticoagulant therapy use or DIC were present, LA should be further investigated in the presence of abnormal waveforms. However, waveform abnormalities were also observed in some cases without LA. Involvements of infection or anticoagulants could not be denied, but this study was underpowered to determine which types of infections or which classes, or doses of anticoagulants were more likely to develop waveform abnormalities, and these issues were to be addressed in future studies.

Further, our multivariate analysis demonstrated positive association between abnormal waveforms and length of APTT. This could be explained that as longer the APTT became, detection of waveform abnormalities became easier. There was significant interaction between them, and it was demonstrated that association between LA and waveform abnormalities became stronger when APTT was less than 60-seconds, although the wide confidence intervals and the limited sample sizes relative to the number of variables in this stratified analysis warranted cautious interpretation. Even so, this result suggested that the impact of clot waveform on the necessity of LA confirmation tests was most pronounced in cases with mildly prolonged APTT. For example, when the patient with APTT < 60 seconds had normal clot waveform and alternative etiologies of prolonged APTT were evident like use of anticoagulants or septic DIC, the priority of LA tests would be lowered. In contract, LA tests should be performed if the APTT was not less than 60 seconds. Our study demonstrated that interpretation of waveform abnormalities should be varied according to the degree of APTT prolongation, which seemed a novel insight.

Unexpectedly, we found that different waveform patterns were observed even within the same patient. Although the limited number of cases in our study precluded the appropriate inference for the underlying reasons, results of coagulation tests were generally influenced by preanalytical factors such as inappropriate blood collection volume, temperature, and storage time.^25^ We could only conclude that in cases in which interpretation based on a single waveform analysis was inconclusive, repeated measurements on different timings would be advisable.

In order to avoid the bias associated with these limitations, larger prospective studies would be desirable. Although other previous studies focused on objective values attained from clot waves such as deceleration/acceleration time ratio,^13^ our study focused solely on visual findings, which might compromise objectivity to some extent. Use of such sophisticated mathematical methods would help detect otherwise overlooked slight abnormalities, but it would be difficult to analyze these complicated parameters for every patient with prolonged APTT in routine clinical practice, especially in community hospitals like ours. It was true that classification by visual findings had some ambiguity, but it was far simpler and more useful in everyday patient care.

In summary, our study elucidated that not only atypical peak pattern, but also other abnormal waveforms could predict the presence of LA, and CWA appeared most useful in patients with mild-to-moderate APTT prolongation, although comprehensive evaluation including LA tests remained essential regardless of waveform analysis in patients with extremely prolonged APTT. Based on the presence or absence of abnormal clot waveform patterns, it would be possible for clinicians to quickly and appropriately determine which additional tests were necessary and which were unnecessary.

### Support

This study was not supported by any funding. There was no any other competing support in relation to this work.

### Other disclosures

There were no other relevant associations that might reasonably represent or create the perception of a COI related to this work.

## Authors’ contributions

KE and MT collected and analyzed the data. KE wrote the manuscript. KF and TT supervised the analyses and helped writing the manuscript.

## Funding

All the authors declared that we had no relevant financial interests.

## Conflicts of interest

The authors declare no have conflicts of interest.

## Data Availability

The data that support the findings of this study are available from the corresponding author upon reasonable request.
