# Supplementary material for: Different patterns of activated partial thromboplastin time (APTT) clot waveforms predicting the presence of lupus anticoagulant
Source: Clinics (Sao Paulo). 2026 Sep 10;81:101136. doi: 10.1016/j.clinsp.2026.101136 (PMC13586575; doi:10.1016/j.clinsp.2026.101136)
Supplement: Supplementary file 1 [file mmc1.docx]

CLINICS-D-25-02107_Supplementary Material

**Supplementary Table 1** Clinical characteristics of first samples of each patient.

|  | **Normal** | **Atypical peak** | **LBBB-like** | **S-shaped** | **p-value** |
| --- | --- | --- | --- | --- | --- |
| **Age** | 80 (69‒89) | 72 (67‒81) | 71 (54‒83) | 76 (60‒81) | 0.35 |
| **APTT** | 60.7 (53.5‒73.9) | 74.3 (59.3‒111) | 58.4 (53.1‒68.9) | 75.9 (54.4‒89.6) | 0.075 |
| **Machine** |  |  |  |  | 0.01 |
| CS-2500 | 75 | 1 | 5 | 7 |  |
| CS-5100 | 49 | 10 | 6 | 6 |  |
| **Gender** |  |  |  |  | 0.81 |
| Female | 61 | 4 | 6 | 7 |  |
| Male | 63 | 7 | 5 | 6 |  |
| **Etiology** |  |  |  |  | <0.01 |
| LA | 2 | 3 | 2 | 2 |  |
| Coagulopathy | 29 | 2 | 0 | 2 |  |
| CPA | 10 | 0 | 0 | 2 |  |
| Hemophilia A | 0 | 1 | 0 | 0 |  |
| Liver disease | 5 | 1 | 0 | 0 |  |
| Heparin | 32 | 0 | 1 | 0 |  |
| Thrombin inhibitor | 5 | 0 | 0 | 0 |  |
| Warfarin | 8 | 0 | 2 | 0 |  |
| Xa inhibitor | 18 | 1 | 2 | 1 |  |
| Others | 1 | 0 | 0 | 0 |  |
| Unknown | 14 | 3 | 4 | 6 |  |
| **Group** |  |  |  |  | <0.01 |
| Group 1 | 117 | 1 | 7 | 0 |  |
| Group 2 | 7 | 10 | 4 | 13 |  |

LA, Lupus Anticoagulant; CPA, Cardiopulmonary Arrest.

**Supplementary Table 2**

|  | **Age** | **Gender** | **Test 1** | **Test 2** | **Test 3** | **Test 4** |
| --- | --- | --- | --- | --- | --- | --- |
| **LA^a^2** | 47 | Female | LBBB |  |  |  |
| **LA^a^3** | 57 | Female | Atypical |  |  |  |
| **LA^a^4** | 59 | Female | LBBB | Atypical^a^ |  |  |
| **LA^a^5** | 66 | Female | Atypical | S-shape^a^ |  |  |
| **LA^a^6** | 72 | Male | Atypical |  |  |  |
| **LA^a^7** | 80 | Female | S-shape |  |  |  |
| **LA^a^9** | 92 | Female | S-shape | S-shape^a^ |  |  |
| **LA^a^1** | 26 | Female | Normal | Normal | Normal | S-shape |
| **LA^a^8** | 84 | Male | Normal^c^ | Normal^c^ | S-shape | Normal |
|  |  |  |  |  |  |  |
|  | **Test 5** | **Test 6** | **Test 7** | **Test 8** | **Test 9** |  |
| **LA^a^1** | LBBB | Normal | Normal | S-shape | LBBB^b^ |  |
| **LA^a^8** | S-shape | Atypical | S-shape |  |  |  |

^a^ These samples were attained in similar conditions compared to first samples.

^b^ Catastrophic APS was diagnosed between test 8 and test 9. Test 9 was attained after start of heparinization.

^c^ These two samples were attained before discontinuing edoxaban. LA was confirmed after that. Prolongation of APTT was not improved, but abnormal waveforms became visible.

**Supplementary Table 3**

|  |  | **Excluded samples (n=28)** | **Included samples (n=271)** | **p-value** |
| --- | --- | --- | --- | --- |
| **Age** |  | 82 (70‒88) | 80 (66‒88) | 0.576 |
| **APTT** |  | 155.9 (151.6‒163.5) | 62.3 (54.4‒80.8) | <0.01 |
| **Machine** | CS2500 / CS5100 | 15 / 13 | 149 / 122 | 1 |
| **Gender** | Female / Male | 14 / 14 | 138 / 133 | 1 |
| **Etiologies** |  |  |  | 0.272 |
|  | LA | 6 | 26 |  |
|  | CPA | 3 | 12 |  |
|  | Hemophilia A | 0 | 1 |  |
|  | Liver disease | 0 | 13 |  |
|  | Drug-induced |  |  |  |
|  | - Heparin | 11 | 68 |  |
|  | - Thrombin inhibitor | 0 | 5 |  |
|  | - Warfarin | 1 | 15 |  |
|  | - Xa inhibitor | 0 | 32 |  |
|  | Coagulopathies by infection | 2^a^ | 29 |  |
|  | Other coagulopathies |  |  |  |
|  | -Massive bleeding | 0 | 11 |  |
|  | -Oncologic DIC | 0 | 3 |  |
|  | -Infectious + oncologic DIC | 1^b^ | 6 |  |
|  | -Other etiologies | 1^c^ | 5 |  |
|  | Unknown | 3 | 45 |  |

^a^ One patient had acute pancreatitis, and the other had intraabdominal infection secondary to gastrointestinal perforation.

^b^ This patient had pneumonia.

^c^ This patient had congenital factor XII deficiency.

LA, Lupus anticoagulant; CPA, Cardiopulmonary Arrest; DIC, Disseminated Intravascular Coagulopathy.
